# The Opportunity of Clinical Pathology

**Published:** 1906-04-14

**Authors:** 


### The Hospital

A Journal of

The Medical Sciences and Hospital Administration.

Vol. XL.—No. 1,020. SATURDAY, APRIL 14, 1906.

### The Opportunity of Clinical Pathology.

In an address recently delivered to the Bristol Medical Society, Mr. Mayo Robson has drawn attention to a question which is closely related to the art of exact diagnosis and to the progress of scientific medicine. The substance of his argument is that the methods and achievements of modern pathology have resulted in the discovery of numerous facts which demand recognition in everyday clinical work; that the detection of such facts, and an estimate of their significance in any individual case, are only possible to those who have enjoyed an adequate training and experience in the laboratory; and that work of this order ought not to be pursued as a detached investigation, but should be associated with a knowledge of the clinical facts, and should receive fees adequate to its importance and responsibility. These propositions, it may be allowed, already receive a more or less general theoretical assent, but they are by no means commonly adopted in practice. Nor will they be adopted until the changed position of pathology in relation to practical medicine and surgery is more widely and keenly recognised. It still remains true that to many pathology is mainly a discussion of the facts of the deadhouse, and is in essence identical with morbid anatomy. That, it is true, was its original foundation and origin, and even the improvement of the microscope and the discovery of the differentiating value of staining agents hardly brought it closer to the living work of the physician and surgeon. Yet, fundamentally, pathology is a knowledge of morbid processes, and the facts recognised on post-mortem examination are merely one of several sources from which such knowledge is to be gained. And it is the recognition and enlargement of other sources which have at the present day made pathology something more than a contemplation of the ruin brought by disease, and have brought it into close, even into vital relationship with the healing work of the medical practitioner. It is sufficient to mention the various methods of examining the blood, the more elaborate chemical investigations of the urine and other fluids, the analysis and microscopic study of the fæces, and the wide applications of bacteriology, to show how in its modern sense pathology offers the most direct assistance to those on whom fall the responsibilities of diagnosis and treatment. Equally it must be allowed, even though the admission is made not without regret, that much of the work involved in these investigations demands an amount of time and attention inconsistent with the claims of clinical practice—that, in short, it necessitates the recognition of yet another order of specialists. This is the actual position, and from it there is no escape. Even those who recognise it grudgingly may find some consolation; for it is morally certain that the advances in the successful practice of the healing art already due to laboratory methods are but the beginning of a great movement which in the future is as likely to revolutionise therapeutics as it is to aid diagnosis. If this general position is allowed it is obvious that where clinical acumen and the ordinary methods of physical examination leave only an uncertain or ambiguous conclusion—and no one will deny that this is not an infrequent experience—the diagnostic possibilities may yet be considerable by the application of laboratory methods. The question of growing importance is, By whom are these to be applied, and how are the results to be co-ordinated with the other facts of the case? The latter point is one of much moment; for both experience and the claims of scientific method forbid the view that laboratory examination, any more than any other limited and exclusive line of investigation, can safely be considered apart from other facts in the solution of a complex clinical problem. It is by a study of all the facts that a correct conclusion is alone possible. From all this it seems to follow that the clinical pathologist is in the immediate future likely to take an increasingly active part in the practical work of medicine and surgery, and that the maximum value of his work is to be obtained, not by restricting him to the mere preparation of a laboratory report on specimens duly submitted, but rather by associating him in full consultation on all the facts with the responsible clinical observer. If this practice becomes the rule it will mean that the clinical pathologist gains not only the dignity and financial recognition to which his work entitles him, but also that he pursues the work under the influence of a due responsibility. To the attainment of these desirable ends, however, it will be essential that laboratory practice, like other branches of practice, shall have a distinctly personal undertaking and guarantee. Examinations deputed to others of unknown competence, reports boasting only a formal signature, and the advertisement of a commercial price list, will not secure either confidence or recognition. If clinical pathology is to have its full opportunity its exponents must recognise the necessity, not only of scientific methods, but also of personal values.

